# X-ray Photospectroscopy and Electronic Studies of Reactor Parameters on Photocatalytic Hydrogenation of Carbon Dioxide by Defect-Laden Indium Oxide Hydroxide Nanorods

**DOI:** 10.3390/molecules24213818

**Published:** 2019-10-23

**Authors:** Joel Y. Y. Loh, Nazir P. Kherani

**Affiliations:** 1 Department of Electrical and Computing Engineering, University of Toronto, Toronto, ON M5S 3G4, Canada; joel.loh@mail.utoronto.ca; 2 Department of Material Science and Engineering, University of Toronto, Toronto, ON M5S 3E4, Canada

**Keywords:** indium oxide, reverse water gas shift, X-ray photospectroscopy, hall mobility

## Abstract

In the study reported herein, glovebox-protected X-ray photoelectron spectroscopy (XPS) and in situ Hall charge carrier measurements provide new insights into the surface physical chemistry of gaseous H_2_, CO_2_, and H_2_+CO_2_ combined with nanostructured In_2_O_(3−x)_(OH)_y_ nanorods, which ensue under photochemical and thermochemical operating conditions. Heterolytic dissociation of H_2_ in H_2_-only atmosphere appears to occur mainly under dark and ambient temperature conditions, while the greatest amount of OH shoulder expansion in H_2_+CO_2_ atmosphere appears to mainly occur under photoilluminated conditions. These results correlate with those of the Hall measurements, which show that the prevalence of homolytic over heterolytic dissociation at increasing temperatures leads to a steeper rate of increase in carrier concentrations; and that H_2_ adsorption is more prevalent than CO_2_ in H_2_+CO_2_ photoillumination conditions.

## 1. Introduction

Photocatalysts are useful tools in the reduction of CO_2_ under solar radiation to generate sustainable fuels [1,2,3]. The thermal dehydroxylation of In(OH)_3_ at 500 °C yields the cubic bixbyite In_2_O_3_ polymorph, the crystal structure of which is based on fluorite, with 25% of the oxide ions missing from the lattice. By controlling the thermal profile used for the dehydroxylation of the In(OH)_3_ precursor, a partially dehydroxylated indium oxide hydroxide is obtained, in which the bixbyite structure is retained [4,5,6,7,8]. By quantifying the amount of water eliminated from the lattice of In(OH)_3_ by thermal gravimetric analysis, the stoichiometry of the oxide and hydroxide groups in the obtained material can be established according to the balanced reaction equation, with the following intermediate stochiometric composition: 2In(OH)_3_ → In_2_O_2x_(OH)_(6−x)_ + 2×H_2_O → In_2_O_3_ + 3H_2_O (~300 °C). The intermediate indium oxide hydroxide product at 250 °C In_2_O_2x_(OH)_(6−4x)_, denoted In_2_O_(3−x)_(OH)_y_, thus, contains hydroxide groups and coordinately unsaturated indium sites, denoted InOH••In. These sites behave as a Lewis base and a Lewis acid individually, but when coupled together can be viewed as a metastable surface frustrated Lewis pair (SFLP) site [9,10]. Oxygen vacancies also exist in the lattice of In_2_O_(3−x)_(OH)_y_, which serve to increase the Lewis acidity of adjacent indium sites. These defects, both coupled and uncoupled, all play pivotal roles in catalyzing the light-assisted reverse water gas shift (RWGS) reaction [11]: CO_2_ + H_2_ → CO + H_2_O. Depending on the ratio of H_2_ to CO_2_ pressure fraction, the selectivity of the RWGS can range from CH_4_ to CO or CH_3_OH. The main product of the 1:1 pressure ratio of H_2_:CO_2_ on the In_2_O_(3−x)_(OH)_y_ catalyst is CO, with a typical yield range of 20–120 ppm/g.cat over a reactor temperature of 100–200 °C, with minor CH_4_ yield. 

For amplification, the first step in the RWGS catalytic cycle (CO_2_ + H_2_ → CO + H_2_O) is purported to involve heterolysis of H_2_ on the SFLP to form InOH_2_••InH, comprised of a proton bound to the hydroxide Lewis base site and a negatively charged hydride to the coordinately unsaturated Lewis acid indium site. The rationale for the heterolytic splitting of H_2_ is due to the strong charge polarization effect of the indium site and OH site [12,13,14]. In general, various polar metal oxides, such as MgO and CeO_2_ [15,16,17] were shown to have strong polarization between the metal and oxide sites, which would heterolytically split H_2_; in In_2_O_(3−x)_(OH)_y_ the polarization is induced by the presence of the oxygen vacancy between the indium and OH sites. In the rest of the cycle, an intermediate formate carbonate species is generated on the InOH_2_••InH supersite, before decomposing into CO and H_2_O from the C–OH_2_In end and the O–HIn end. In the work described herein, quasi-operando X-ray photoelectron spectroscopy (XPS) probes the interaction of H_2_, CO_2_, and H_2_+CO_2_ with the surface of pristine nanostructured In_2_O_(3−x)_(OH)_y_. The difference between an ex situ XPS measurement and the following study is that the reactors with the samples contained within were directly transferred into the glovebox after the various gas treatments, and the samples were transferred to the XPS within the argon-filled glovebox. Hence, the surface condition reflects that of the stable chemisorbed species after reactor treatments. These results provide a deeper understanding of the chemistry in the ground and excited states relevant to the RWGS reaction. We also conducted in situ Hall carrier property measurements under the various gas reactant atmospheres in dark and photoillumination conditions, which allowed us to associate the chemisorption of the reactants to the electronic properties of the In_2_O_(3−x)_(OH)_y_ nanorods. 

## 2. Results and Discussion

**Figure 1:**
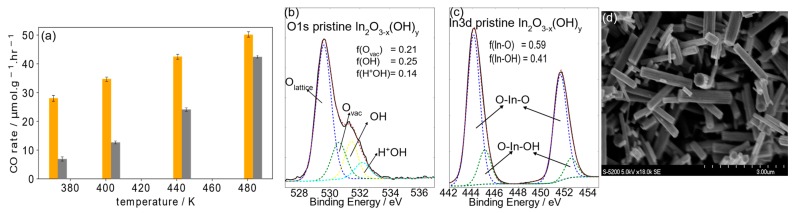
(**a**) The CO production rate with the In_2_O_(3−x)_(OH)y catalyst under white light at 1:1 pressure ratio of H_2_:CO_2_ gas atmosphere, and under dark conditions with increasing reactor temperatures. (**b**,**c**) The O1s and In3d XPS spectrum of In_2_O_(3−x)_(OH)_y_ nanorods as prepared. (**d**) SEM topographical image of In_2_O_(3−x)_(OH)_y_ nanorods.

The CO production rate from the reverse water gas shift reaction on In_2_O_(3−x)_(OH)_y_ nanorods shows a significantly greater enhancement under white light photoillumination of 140 mW/cm^2^ than under dark conditions, with the increase in CO rate diminishing with higher temperatures. The maximum rate under white light and reactor temperature of 210 °C is 50.2 µmol/g.cat/hour, with a pseudo activation energy of 10.9 kJ/mol/K, which is smaller by a factor of 0.46 than the activation energy under dark conditions. The XPS O1s core-level binding energies of In_2_O_(3−x)_(OH)_y_ nanorods in a vacuum can be resolved into four peaks (limited to a maximum of 1.4 eV FWHM), assigned to lattice oxide around ~529.5 eV, oxygen vacancy ~530.5 eV, hydroxide ~532 eV, and protonated OH groups at ~532.5 eV (Figure 1b). The line widths of these O1s peaks are often broad and asymmetric because of multiple site occupancies of oxide lattice O_lattice_ (40% of total species), oxygen vacancies O_vac_ (21%), and hydroxide OH type species (39%) on the surface of In_2_O_(3−x)_(OH)_y_. The effective positive charge of the proton bonded to the oxide site of the hydroxide causes the O1s ionization potentials to shift to higher energy than the lattice oxide, while the protonated OH species (H^+^OH) (14% of total species) arising from ambient moisture during synthesis preparation [18,19] causes a positive shift for some of the OH groups, which all result in a broad OH shoulder. In addition, the presence of an oxygen vacancy in the oxide coordination sphere of indium enhances binding of the remaining oxides to the indium, which is manifest as a shift to higher energy of the O1s ionization potentials. We note that all In3d peaks were normalized between the various conditions, and the same normalization factor applied towards the O1s and C1s spectra. The corresponding In3d XPS results for nanostructured In_2_O_(3−x)_OH_y_ are shown in (Figure 1c). The In3d^3/2^ spin orbit component is found to have an ionization potential around 444 eV. Its line width contains contributions from In–OH and In–O species, of which the 41% ratio of OH-type species over the total is similar to that of the O1s spectrum.

**Figure 2:**
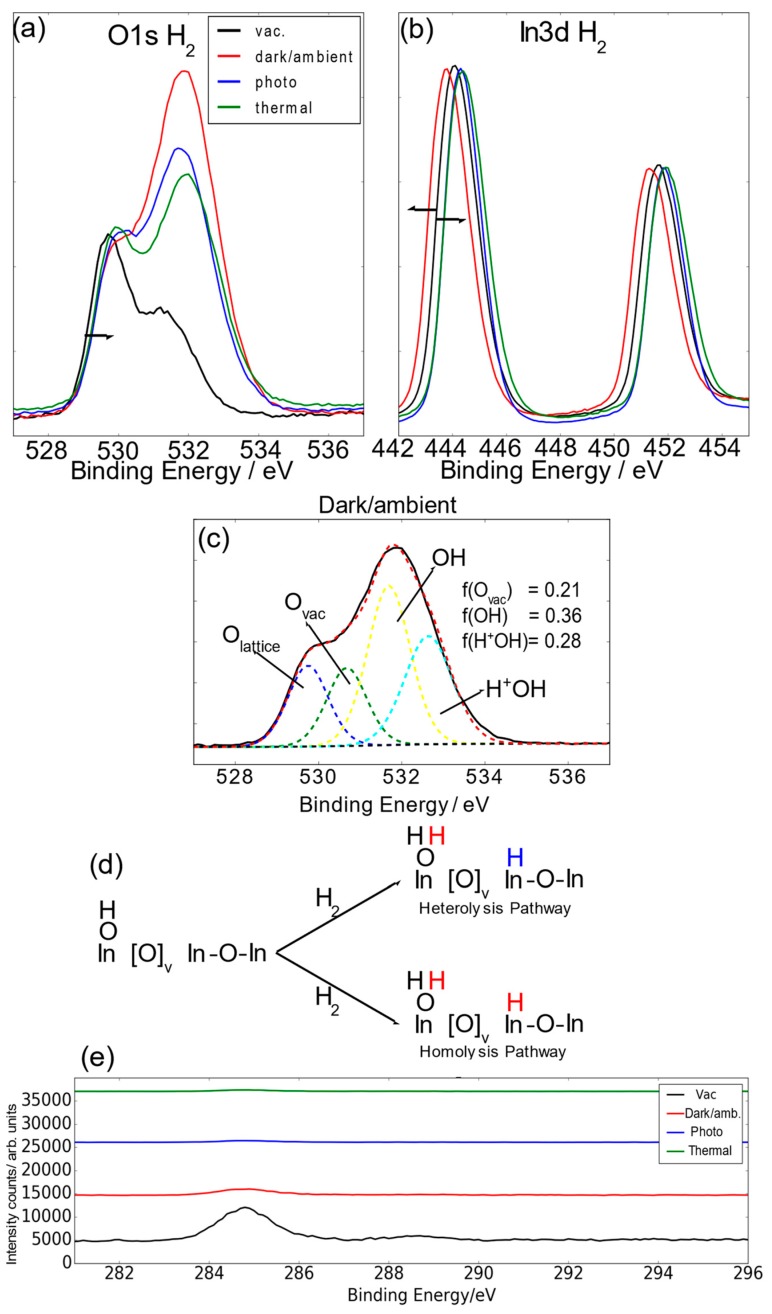
(**a**,**b**) The O1s and In3d X-ray photoelectron spectroscopy (XPS) spectra of In_2_O_(3−x)_(OH)_y_ nanorods in H_2_ under various reactor conditions in comparison with the pristine surface shown in Figure 1. (**c**) The XPS deconvolution of the O1s spectrum under H_2_ dark ambient conditions into various oxygen species. (**d**) Heterolysis and homolysis dissociation of H_2_ molecules over a surface frustrated Lewis acid–base pair site. (**e**) C1s spectra of In_2_O_(3−x)_(OH)_y_ under H_2_ in various conditions.

On exposure of In_2_O_(3−x)_(OH)_y_ nanorods to H_2_ at under dark and ambient room temperature (dark ambient) conditions a dramatic increase in the intensity and shift to high energy of approximately 0.5 eV occurs for the O1s binding energy of the hydroxide (Figure 2). This observation flags the dissociation of H_2_ on the surface of In_2_O_(3−x)_(OH)_y_. Noticeably, the fraction of O_vac_ remains similar for all conditions under H_2_, indicating that the new hydroxide formation associated with ~532.2 eV is mostly hydrogen adatoms on lattice oxide sites to form bridging OH groups. Concomitantly the In3d^3/2^ peak undergoes a notable shift to lower energy implying the indium is experiencing a lower effective nuclear charge (Figure 2b). This behavior arises from a coordinately unsaturated Lewis acidic surface indium site bonded to a highly nucleophilic hydride formed by heterolysis of H_2_ on a SFLP site, which must dominate the opposing effect of protonation (Figure 2d). In addition, homolysis of H_2_ on hydroxide surface sites adjacent the oxygen vacancy sites, protonates these OH groups. The associated injection of charge-balancing electrons into defect indium sites cause the In3d^3/2^ peak to shift to lower energy, providing it overrides the countering effect of protonation. With H_2_ exposure under photoillumination, the intensity of the OH shoulder shrinks below its dark ambient value. Since the OH shoulder also experiences a small binding energy decrease from dark ambient to phototreatment, this indicates that H^+^ adatoms on OH groups are removed, and less so for H^+^ adatoms on lattice oxide sites. Heating at 150 °C causes further shrinkage of the intensity of this OH shoulder. The OH shoulder binding energy is similar to that of the H_2_ dark ambient condition, indicating that more H adatoms on lattice oxides are removed and less so for H adatoms bound on OH groups. These observations signal the photochemical and thermochemical induced loss of H_2_O from protonated hydroxide and indium hydride sites which indicates that indium hydride sites are photo and thermally unstable. Additionally, with introduction of H_2_ there is a diminishment of the intensity of C1s peaks indicating removal of C–C surface contaminants on the In_2_O_(3−x)_OH_y_ nanorods (Figure 2e). Further information on the deconvolution of the O1s spectra are shown in the supplementary section. 

**Figure 3:**
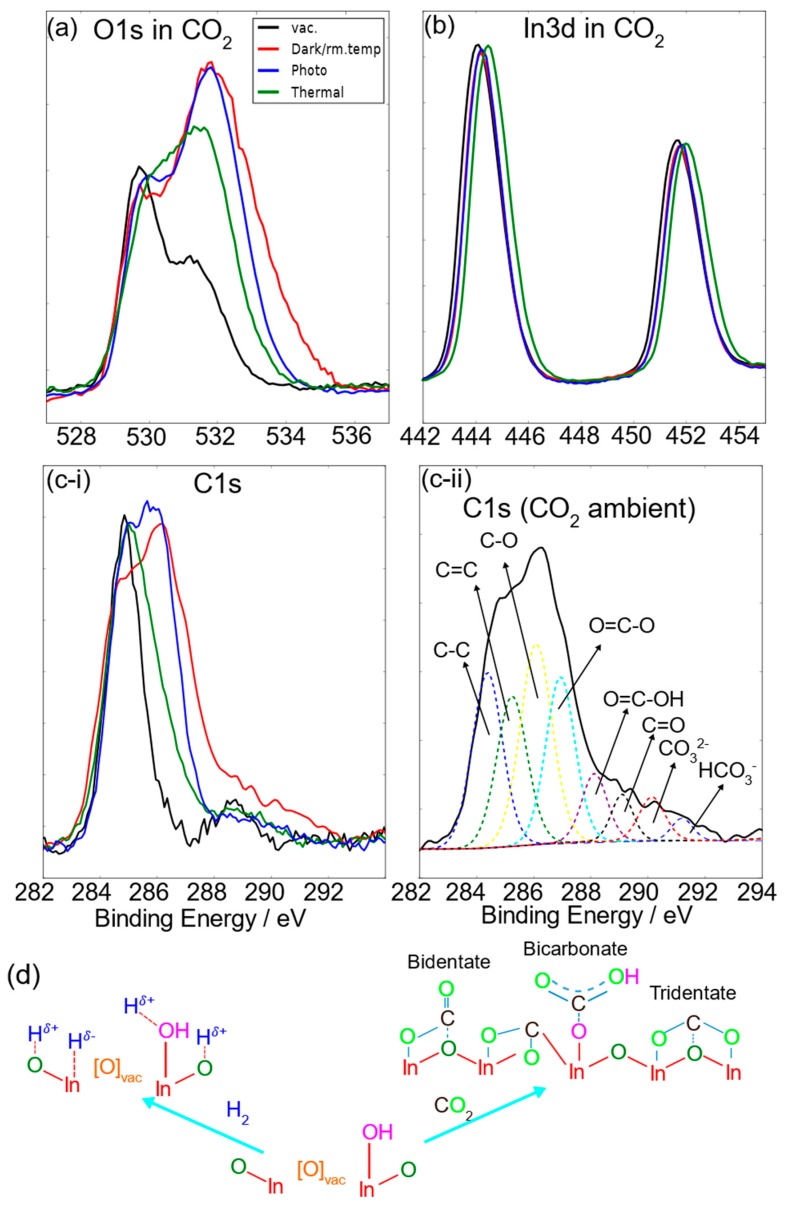
(**a**–**c**) The O1s, In3d, and C1s XPS spectra of In_2_O_(3−x)_(OH)_y_ nanorods in CO_2_ under various reactor conditions in comparison with the pristine surface shown in Figure 1. (**d**) The two adsorption paths for a defective In_2_O_(3−x)_(OH)_y_ surface for H_2_- and CO_2_-only atmospheres. For bidentate configurations, the adsorption energy was determined [12] to be −0.70 eV; tridentate had −1.25 eV adsorption energy; and CO_2_ absorbed on an oxygen vacancy with an adsorption energy of −0.61 eV.

The XPS study of O1s, In3d, and C1s surface species arising from the interaction of the acidic CO_2_ gas probe under dark ambient conditions on pristine, nanostructured In_2_O_(3−x)_(OH)_y_ is rich in detail (Figure 3). In the C1s XPS, the growth of new peaks in the region of 285–288 eV signals the adsorption or reaction of CO_2_ with oxygen vacancy, hydroxide, and coordinately unsaturated indium surface sites. Monodentate configurations are more favorable on surfaces with high basicity and bidentate, or tridentate configurations on surfaces with low basicity. It has been shown that on In_2_O_3_ surfaces, tridentate and bidentate configurations are favourable [12]. For example, two types of tridentate configurations are energetically favorable, where in one case, the carbonate bridges two In–O–In chains to form a cross-wise configuration with an absorption energy of −1.14 eVm; in the other case, a tridentate configuration, where the CO_2_ is in the same plane as In–O–In, the absorption energy is −1.25 eV. A conventional bidentate configuration of carbonate has an absorption energy of −0.70 eV. The significant amount of C–O and O=C signals indicate that bidentate and tridentate carbonate configurations are dominant, while the increased O=C–O/O=C–OH signal indicates bicarbonate products from CO_2_ reactions with OH groups. This model receives support from the corresponding O1s XPS, where adsorption or reaction of CO_2_ in general with In_2_O_(3−x)_OH_y_ increases the effective nuclear charge of the oxide, seen as a small shift of the O1s to higher energy. The hydroxide shoulder around 532 eV increases in height and widens in dark ambient CO_2_ atmosphere, indicating highly positively charged oxide species as a result of the exposure of carbonate species to the O1s spectrum, with C–O species in the range of 531.5–532.0 eV, C=O in the range of 532.0–533.0 eV, and O=C–O species from 533.3–533.6 eV. The effect of light removes the higher binding energy component in the O1s spectrum, indicating surface bicarbonate fragmenting to CO_2_ and H_2_O. The remaining bidentate and tridentate carbonates induce a higher OH shoulder than that of the vacuum state. The removal of bidentate carbonates to leave behind carbonate configurations with dominant C–O bonds under thermal treatment narrows the OH shoulder and shifts the O_Lattice_ subpeak to a higher binding energy, which indicates that either tridentate carbonates or oxygen vacancies may have been filled by carbon dioxide to form species with C–O bonds. This indicates that only such carbonate species persist under thermal treatment. 

**Figure 4:**
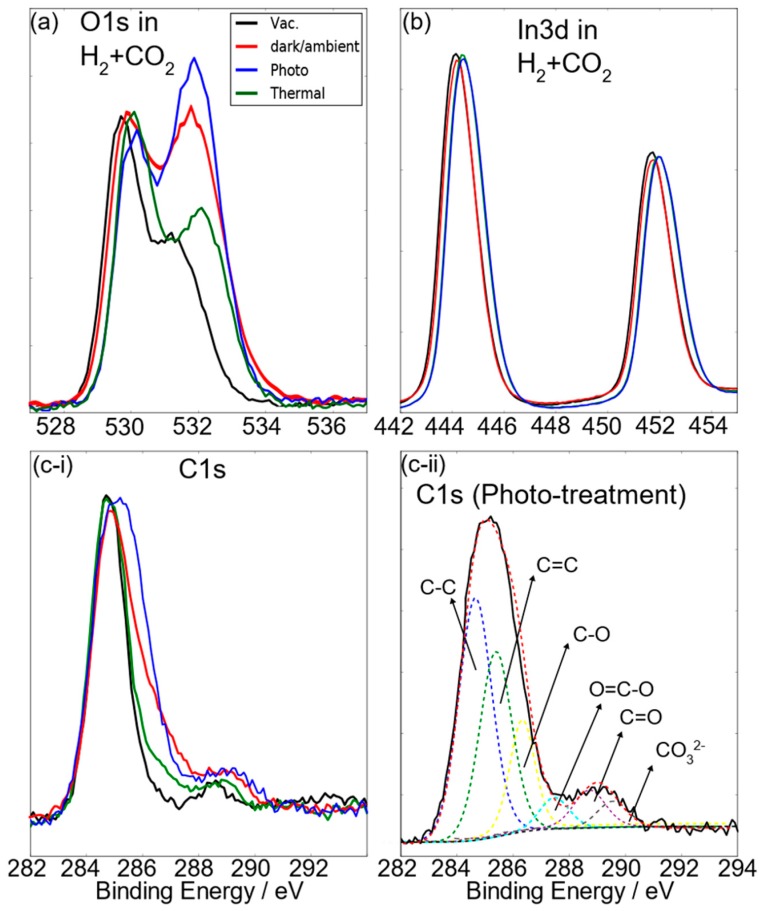
(**a**–**c**) The O1s, In3d, and C1s XPS spectrum of In_2_O_(3-x)_(OH)_y_ nanorods in H_2_+CO_2_ under various reactor conditions in comparison with the pristine surface shown in Figure 1. (**c-ii**) shows the various carbon species in the C1s spectrum after H_2_+CO_2_ under photoillumination conditions.

On exposure of nanostructured In_2_O_(3−x)_OH_y_ to both H_2_ and CO_2_ under ambient temperature, thermal, and photo conditions, the O1s XPS spectrum shows a substantial increase in the intensity of the hydroxide shoulder, with a concurrent shift to higher energy of the latter oxide peak, while the In3d XPS spectra show a similarly small shift. The C1s XPS spectra reveal the presence of C–O-containing species generated photochemically from the reaction of H_2_ and CO_2_ to generate a C1s spectra similar to that of CO_2_-only thermal conditions (Figure 4). There is also an increased C1s signal at ~288–289 eV, indicating C=O and O–C=O species. Since the O1s ionization potential shift is greater for photo than for dark ambient conditions, this implies that part of the OH shoulder height increase is due to increased H_2_ dissociation on both OH and O_Lattice_ sites, and part of it is due to increased carbonate formation. The results, thus, suggest that the surface is highly active under photo conditions. H_2_+CO_2_ mix to form a basic surface that enables adsorption of CO_2_ molecules as bidentate and tridentate species, since C–O and O–C=O bonds seem to be the most stable bond formations under photo conditions. This can be linked to the photoexcited valence band holes and conduction band electrons into the hydroxide Lewis base defect near the conduction band edge, and indium Lewis acid defects near the valence band edge. The trapped electron and hole enhance the Lewis acidity and basicity of any indium hydride and protonated hydroxide group in the excited state, compared to the ground state [20,21]. However, one difference between the H_2_ and H_2_+CO_2_ atmosphere is the straddle between the O_lattice_ peak and OH species-type shoulder, where there is a deeper saddle for the H_2_+CO_2_ case but less so for the H_2_ only case, indicating a small O_vac_ fraction decrease from 22% in the H_2_-only condition to 18% in H_2_+CO_2_ photoillumination. Taken together, it is clear that the presence of OH and O_vac_ sites create a strong polarization effect in both homolytic and heterolytic dissociation of H_2_ to form hydride H^-^ adatoms on the neighboring indium sites, H^+^OH sites, and H^+^O sites. The O1s spectra consistently shows a high OH-type peak with an increased binding energy compared to vacuum conditions. H^+^OH and H^+^O sites, thus, persist mostly under photoillumination conditions due to stabilizing effects of the addition of photoexcited holes. In addition, there is also some indication that oxygen vacancies are filled in the CO_2_-only and H_2_+CO_2_ atmosphere. A possible reaction pathway involves carbonate filling of oxygen vacancies to form a bidentate-like carbonate with a C–O bond, with an adsorption energy of −0.61 eV. This is activated by a neighboring In–H hydride to form a formate species, with a small activation energy barrier of 0.15 eV and an exothermic reaction energy of −0.21 eV [12].

**Figure 5:**
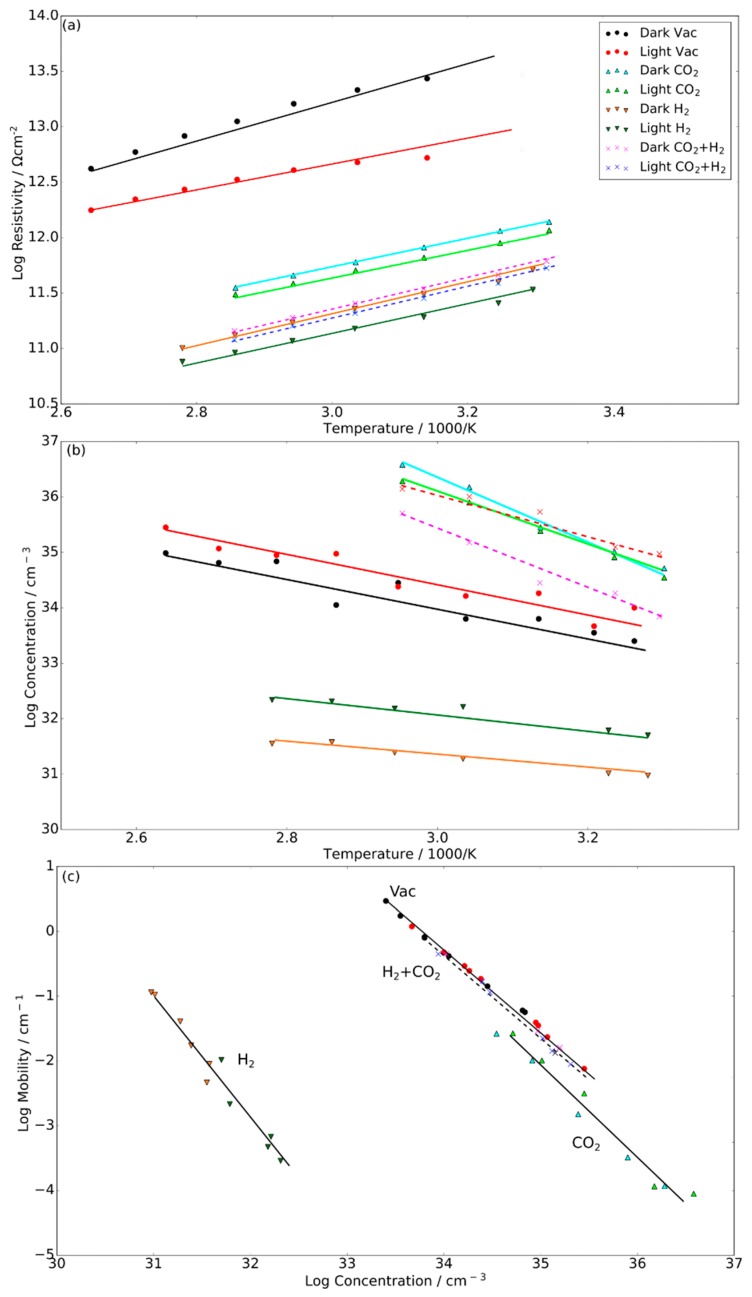
(**a**) Electrical resistivity and (**b**) carrier concentration as a function of temperature, with (**c**) carrier mobility and concentration trends. The results for the vacuum, H_2_, CO_2_, and H_2_+CO_2_ atmospheres are indicated with circle, downside triangle, upside triangle, and cross markers, respectively. Trend lines are shown as a visual guide, with dotted lines representing CO_2_+H_2_ atmospheres.

We now turn to in situ Hall measurements to examine the surface reactivity in greater depth (Figure 5). Generally, electrical resistivity (Figure 5a) decreases with photoillumination, with the greatest decrease occurring in vacuum atmosphere with H_2_, followed by H_2_+CO_2_, and the smallest decrease occurring in vacuum atmosphere for CO_2_. CO_2_ and CO_2_+H_2_ show the smallest photoinduced resistivity decrease of ~10% and ~13%, respectively. For the associated carrier concentration trends, carrier concentrations also follow the trends of the resistivity plot for vacuum and CO_2_, however, for H_2_+CO_2_, there is a distinct difference in the temperature dependence slope, with the slope under photoillumination being greater than that of the dark condition. Furthermore, the carrier concentration for H_2_ dark and photoilluminated conditions are relatively similar in the same order of magnitude, with a steeper temperature dependence under photoillumination than under dark conditions. Generally, carrier mobility decreases under photoillumination, with increased electron–hole pair recombination as a result of carrier concentration increase; hence, there is also a logarithmic linear dependence between mobility and concentration, as shown in Figure 5c. In_2_O_(3−x)_(OH)_y_ in CO_2_ atmosphere has generally low carrier concentrations and lower intrinsic carrier mobilities (extrapolated mobilities at very low concentrations) because of higher electron–hole pair recombination rates. Interestingly, the intrinsic carrier mobilities in vacuum are higher than in the H_2_ or CO_2_ atmospheres, showing that reactant gas chemisorption induces higher recombination rates. The H_2_+CO_2_ atmosphere mobility concentration trend is, however, very similar to that of the vacuum trend. 

The trends shown in Hall measurements can be mostly explained by the aforementioned XPS results. A majority homolytic dissociation of H_2_ injects free electrons through ½H + O_L_ → OH^+^ + *e^−^* and OH + ½H_2_ → H^+^OH + *e^−^*, whereas heterolytic dissociation of H_2_ to form indium hydride generates hole states that can be recombined with any electrons generated from homolytic dissociation. This would explain the sharper increase of carrier concentrations from dark ambient temperature to dark high temperatures, as well as the larger spread of the concentration–mobility trend in comparison to other gas atmospheres. Since the homolytic dissociation of H_2_ under photo condition is fairly similar to that of the 150 °C thermal condition, photoillumination and thermal conditions would likely induce greater homolytic dissociation of H_2_, which can be rationalized in the intersecting trend of increasing carrier concentration with increasing temperature under photo and dark conditions. The increase in carrier concentration as a result of H_2_ dissociation is, however, partly mitigated by the decrease in carrier mobilities, since H^+^OH groups can act as strong electron traps. The trends shown with the CO_2_-only atmosphere indicate that carbonate adsorption depletes free carrier densities and that surface carbonates act as significant carrier scattering sites that increase recombination rates. Since phototreatment induces less carbonate formation, the depletion of free carrier density is reduced, leading to an increase in free carrier density. Furthermore, carbonate formation decreases with temperature, which can explain the increase in concentration with temperature; however, because carbonates are still seen at high temperatures, the increase in carrier density is more gradual. Under H_2_+CO_2_, the mixture of H_2_ dissociation and carbonate formation results in a dynamic carrier population that has both the characteristics of CO_2_- and H_2_-only trends. In dark thermal conditions, homolytic dissociation on the OH group is shown to be more likely than on oxygen lattice sites. As such, the more gradual concentration increase than for H_2_-only group can be linked to lower dissociation of H_2_. In photoillumination condition, while the OH shoulder expansion may indicate either prevalent H_2_ dissociation or prevalent carbonate absorption, the higher carrier concentration compared to that of dark condition indicates that H_2_ dissociation is more favored than carbonate absorption. However, the gradual sloping of the temperature dependence in photoillumination may indicate that carbonate absorption is slightly more favored at higher temperatures under photoillumination. 

## 3. Conclusions

In conclusion, quasi-operando XPS and in situ Hall measurements with the various reactant atmospheres and reactor parameters have provided a new window into the reaction of gaseous H_2_, CO_2_, and H_2_+CO_2_ with nanostructured In_2_O_(3−x)_(OH)_y_ under photochemical and thermochemical operating conditions. While changes in the O1s and In3d core level ionization potentials clearly depict the dissociative adsorption of H_2_ on the surface of nanostructured In_2_O_(3−x)_(OH)_y_, the results clearly distinguish the homolysis from the heterolysis pathway. These results also show the importance of photoillumination and the reason for In_2_O_(3−x)_(OH)_y_ as being a high efficacy photocatalyst, which is to induce greater H_2_ dissociation and carbonate stability than under thermal conditions. The Hall results show the link between the surface compositional changes with electronic activity, with varying temperature and photo-dependences due to the various populations of chemisorbed species. These results show that surface compositions play important roles in the multiple step process of the RWGS, and can be utilized for various photocatalysts and carbon-based nanocomposites [22,23,24]. 

## 4. Materials and Methods

**Synthesis Procedure**: The In_2_O_(3−x)_(OH)_y_ nanorod structures discussed in this paper were fabricated through the following steps: 0.397 g of InCl_3_ was dissolved in 6ml ethanol with stirring. A separate mixture of 2.5 mL NH_3_OH, 7.5 mL ethanol, and 2 mL H_2_O was mixed in the InCl_3_, creating a white suspension. To control for uniform size distributions, the white suspension was immersed in a heated oil bath at 80 °C with stirring for 10 min. The suspension was then centrifuged, excess solvent was removed, and it was subsequently washed with deionized water. This step was done 3 times before a vacuum drying procedure at 70 °C for 12 h. The dried powder was then calcined for 12 h at 250 °C to form the In_2_O_(3−x)_(OH)_y_ phase nanorod structures. The nanorods had an average length of 1800 nm, 13 nm diameter, and Brunauer-Emmett-Teller (BET) derived surface area of 151 m^2^/g. 

**XPS measurements**: The reactor-based quasi-operando XPS measurements were performed with the intention of mimicking the reactor sequence in reducing CO_2_ with H_2_ with this material system. The powder samples were placed on glass slides and a glass pipette, which were used to roll and drag the powders until a visibly smooth film was obtained. Three samples were prepared for each gas condition (H_2_, CO_2_, and H_2_+CO_2_). Each sample was placed in a high vacuum ConFlat flange assembly and sealed with a UV-enhanced transmissive viewport flange using copper gaskets. H_2_ and CO_2_ were introduced at 2 atm pressure after vacuum pumping to ~2 × 10^−5^ mbar. For H_2_+CO_2_, H_2_ was introduced at 2 atm pressure, vented to 1 atm pressure, and CO_2_ was introduced at 1.6 atm pressure. The reactors were then subjected to phototreatment (40 W/cm^2^ intensity white lamp), thermal treatment (150 °C), or control condition (no photoillumination and no thermal treatment) for ~12 h. The nanorod samples were then removed from their reactors inside a grade 5 argon-filled glovebox with O_2_ and H_2_O concentrations of 0.5–0.7 ppm and 0.1–0.3 ppm, respectively. They were then attached to the XPS instrument and loaded directly into the instrument. The instrument was an Thermo Scientific™ ESCALAB™ XPS system (Thermo Scientific, Waltham, MA, USA).

**Hall measurements**: The in situ hall measurements were carried out on a modified Nanometrics Hall system, where the sample box was sealed after applying the contact probes on the sample, which had 4 square gold electrodes 2 mm in width and 500 nm thickness at the corners of the sample film. The electrodes were set 0.5 mm apart. After sealing the sample box, a rough vacuum pumping was applied, after which the gas atmospheres were introduced with a 1.3 atmosphere pressure, and a pressure release valve equilibrated the pressure to 1 atmosphere pressure.

## Supplementary Materials

The following are available online. Figure S1. XPS deconvolution after H_2_ immersion. While the Ovac specie remain relatively constant, the OHT group increase by 144% is significant in H_2_ dark ambient. 26–28% of OHB group is lost in photo/ thermal condition. Figure S2. XPS deconvolution after CO_2_ immersion. Control means dark ambient temperature conditions. For photo condition, there is a significant C–O and O=C–O peak whereas in thermal condition C–O peak increases. Figure S3. XPS O1s deconvolution after H_2_ + CO_2_ immersion. Under dark ambient and photo condition, the OH shoulder expands to nearly 50% of the total O type species. Figure S4. Left figure. C13 CO2 isotope testing by GCMS Agilent 5975C with Mass Spectrometry. The C13O peak is identified at the 1.33 min mark. Right figure, EPR of nanorod powder in quartz capillary tube. To fit the spectrum, the g factors of 3 spin systems with isotropic states are 2.04; 2.116; 2.18 with spin concentration ratios of 0.35:1:0.1 respectively. Figure S5. The UV-Vis spectrum of the In_2_O_3-x_(OH)_y_ powder film, showing the weak absorption tail in the visible which can be associated with band gap deep defects.
