# Supplementary material for: X-ray Photospectroscopy and Electronic Studies of Reactor Parameters on Photocatalytic Hydrogenation of Carbon Dioxide by Defect-Laden Indium Oxide Hydroxide Nanorods
Source: Molecules. 2019 Oct 23;24(21):3818. doi: 10.3390/molecules24213818 (PMC6864452; doi:10.3390/molecules24213818)
Supplement: Supplementary file 1 [file molecules-24-03818-s001.pdf]

Supplementary information for "X-ray Photo-Spectroscopy and Electronic Studies of Reactor Parameters on Photocatalytic Hydrogenation of Carbon Dioxide by Defect Laden Indium Oxide Hydroxide Nanorods"

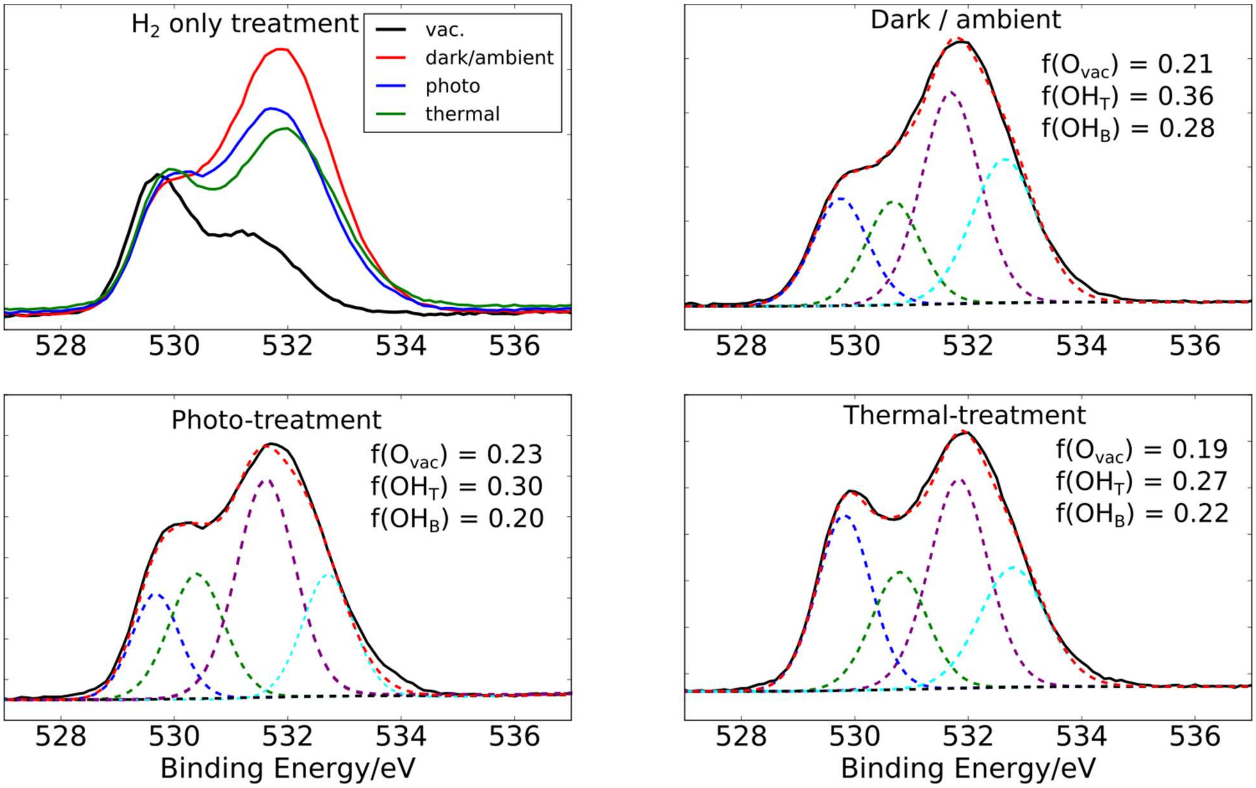

Figure S1. XPS deconvolution after H2 immersion. While the O_{vac} specie remain relatively constant, the OH_T group increase by 144% is significant in H2 dark ambient. 26-28% of OH_B group is lost in photo/ thermal condition.

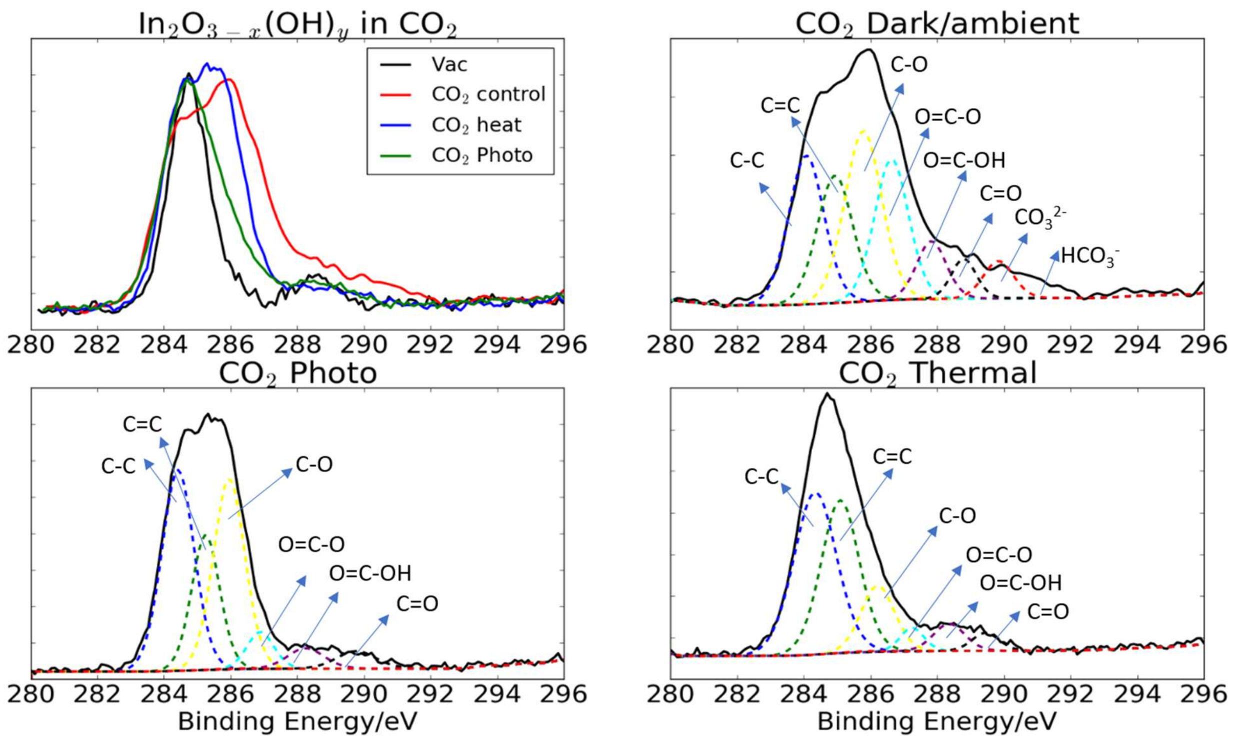

Figure S2. XPS deconvolution after CO_2 immersion. Control means dark ambient temperature conditions. For photo condition, there is a significant C-O and O=C-O peak whereas in thermal condition C-O peak increases.

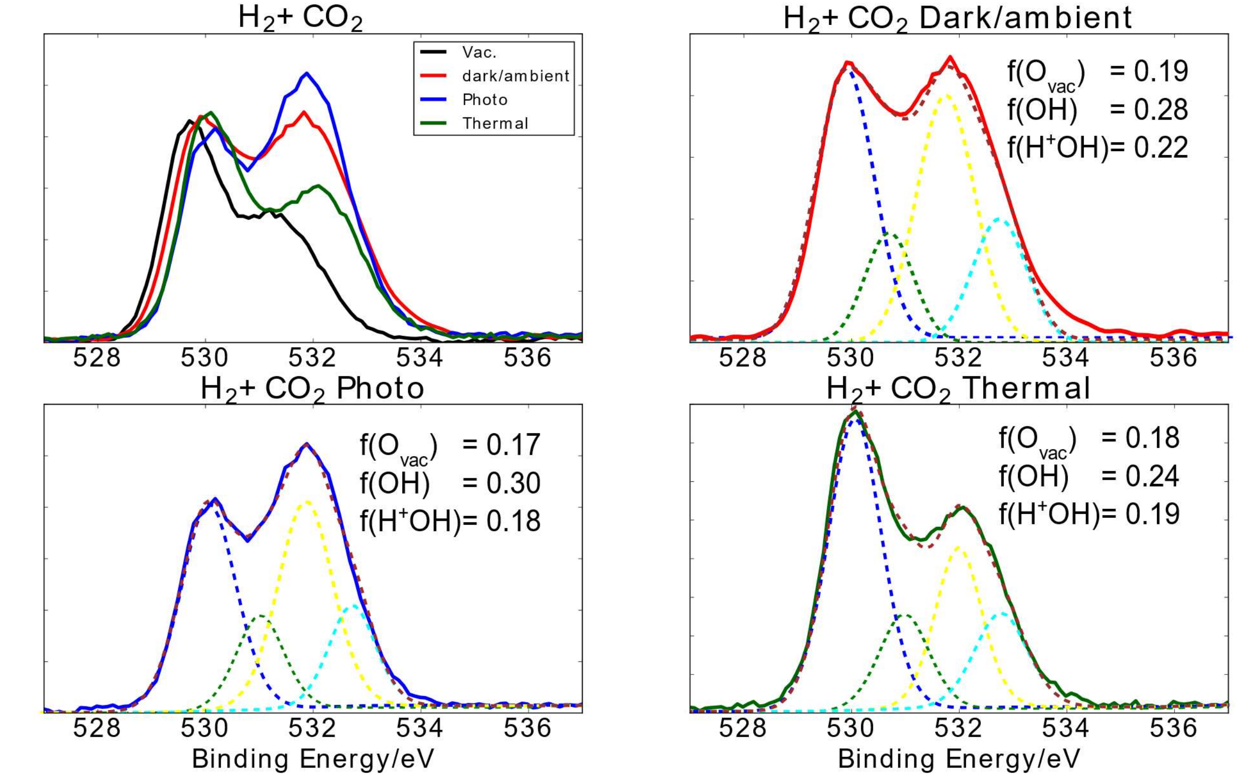

Figure S3. XPS O1s deconvolution after H_2+CO_2 immersion. Under dark ambient and photo condition, the OH shoulder expands to nearly 50% of the total O type species.

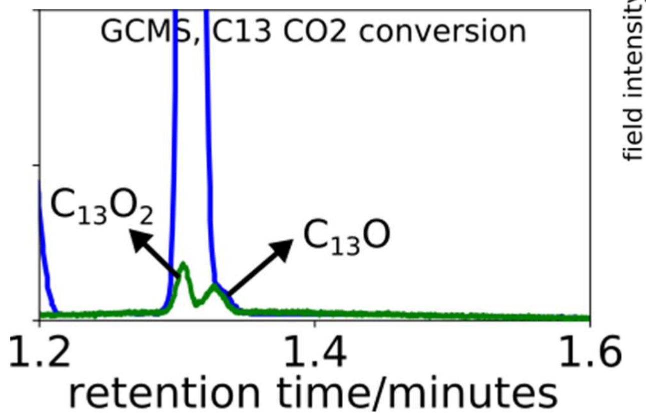

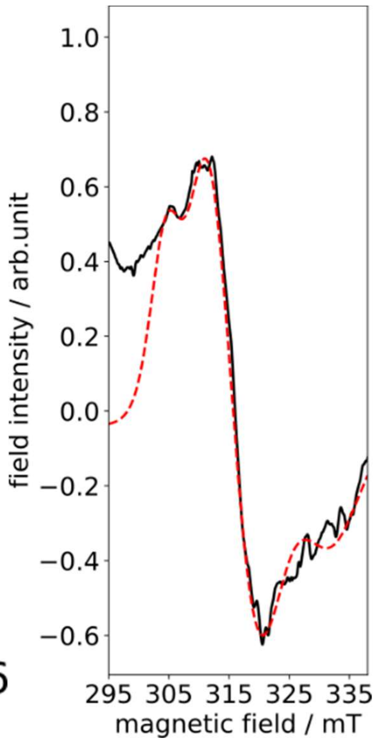

Figure S4. Left figure. C^{13} CO_2 isotope testing by GCMS Agilent 5975C with Mass Spectrometry. The $C^{13}O$ peak is identified at the 1.33 minute mark. Right figure, EPR of nanorod powder in quartz capillary tube. To fit the spectrum, the g factors of 3 spin systems with isotropic states are 2.04 ; 2.116 ; 2.18 with spin concentration ratios of 0.35 :1 : 0.1 respectively.

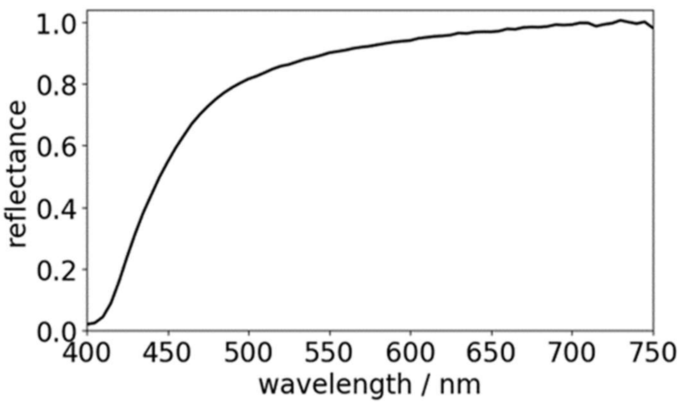

Figure S5. The UV-Vis spectrum of the $In_2O_{3-x}(OH)_y$ powder film, showing the weak absorption tail in the visible which can be associated with band gap deep defects.
